# Signals behind *Listeria monocytogenes* virulence mechanisms

**DOI:** 10.1080/19490976.2024.2369564

**Published:** 2024-07-09

**Authors:** Diana Meireles, Rita Pombinho, Didier Cabanes

**Affiliations:** a Instituto de Investigação e Inovação em Saúde, Porto, Portugal; b Group of Molecular Microbiology, IBMC, Porto, Portugal; c Instituto de Ciências Biomédicas Abel Salazar – ICBAS, Porto, Portugal

**Keywords:** Listeria monocytogenes, Virulence mechanisms, Virulence signals

## Abstract

The tight and coordinated regulation of virulence gene expression is crucial to ensure the survival and persistence of bacterial pathogens in different contexts within their hosts. Considering this, bacteria do not express virulence factors homogenously in time and space, either due to their associated fitness cost or to their detrimental effect at specific infection stages. To efficiently infect and persist into their hosts, bacteria have thus to monitor environmental cues or chemical cell-to-cell signaling mechanisms that allow their transition from the external environment to the host, and therefore adjust gene expression levels, intrinsic biological activities, and appropriate behaviors. *Listeria monocytogenes* (*Lm*), a major Gram-positive facultative intracellular pathogen, stands out for its adaptability and capacity to thrive in a wide range of environments. Because of that, *Lm* presents itself as a significant concern in food safety and public health, that can lead to potentially life-threatening infections in humans. A deeper understanding of the intricate bacterial virulence mechanisms and the signals that control them provide valuable insights into the dynamic interplay between *Lm* and the host. Therefore, this review addresses the role of some crucial signals behind *Lm* pathogenic virulence mechanisms and explores how the ability to assimilate and interpret these signals is fundamental for pathogenesis, identifying potential targets for innovative antimicrobial strategies.

## Introduction

Pathogen adaptability is pivotal to the infection outcome and is determined by bacterial factors allowing the establishment and progression of the infection. Importantly, the tight and coordinated regulation of these virulence genes is crucial and strategic for their expression in specific time-frames, within particular tissues, or by bacteria subsets. Moreover, the untimely expression of virulence factors represents a major fitness burden that can be detrimental to a successful survival strategy, once bacteria do not express virulence factors homogenously in time and space. Infections caused by Gram-positive bacteria are challenging and still remain a major public health. *Listeria monocytogenes* (*Lm*) is a major Gram-positive intracellular foodborne pathogen which causes a life-threatening infection, listeriosis, acquired from the ingestion of contaminated food. It is the most severe human systemic infection among zoonotic diseases under EU surveillance. It can be particularly severe in specific high-risk groups, including pregnant women, elderly and immunocompromised people. Despite the availability of antibiotics-based treatment, listeriosis remains the most frequent cause of death due to the consumption of contaminated food in Europe, with the higher proportion of hospitalized cases (~96%) and a case mortality rate of ~18.1%.^1,2^ *Lm* is ubiquitously found in a wide range of environments, such as soil, water, animal feces and vegetation, being constantly exposed to a number of stress adaptations both throughout its saprophytic life and transiting to infect human tissues. Once inside the host, it is capable to invade and colonize the gut, transverse the intestinal barrier and disseminate, establishing a systemic infection, and replicating within target organs.^3,4^ Importantly, the expression of virulence factors is directly dependent on the *Lm* ability to detect and process signals from the surrounding environment adjusting its physiology accordingly.

The coordinated phenotypic expression of *Lm* virulence factors and the underlying mechanisms of their regulation provide a competitive advantage for the bacterial community in different contexts, such as biofilm formation, host infectionor antibiotic resistance. This review focuses on the role of different signals behind *Lm* virulence gene expression and how they impact bacterial communication throughout host infection and therefore pathogenesis. Since the successful host colonization by pathogens is conditioned by the signals they integrate, it is crucial to understand how these signals act to further modulate bacterial colonization and therefore control disease. Importantly, most of the signals required to control virulence mechanisms are transversal to different pathogens. This review provides an overview of both the signaling molecules generated by *Lm* itself for its own profit and the nutritional and physical external signals that control bacterial virulence (Table 1).

**Table 1.** Summary of signals sensed by *Lm* to modulate virulence.

Category	Signal	Function/related processes	Associated genes	Refs
*Lm* signaling molecules	S-adenosylmethionine (SAM)	Involved in PrfA regulation via SreA and SreB riboswitches	metK, *lmo2417*, *lm0135, sreA-B*	5–11
	AI-2	Involved in S-adenosylhomocysteine (SAH) detoxification		12
	S-ribosylhomocysteine (SRH)	Involved in biofilm formation and production of AI-2		9
	Autoinducer peptide (AIP)	Triggers the induction of the Agr system	*agrD*	13
	Peptide-pheromone pPplA	Signals the presence within the host vacuole and facilitates vacuolar escape by promoting PrfA activation	*lmo2637*	14
	c-di-AMPs	Maintains intracellular GTP levels required for CodY expression		15–17
	Glutathione	Mediates PrfA activation		18–20
	Vitamin B_12_ /cobalamin	Impairs *aspocR* and *rli55* transcription to control propanediol and ethanolamine metabolism	rli55, *eutV*, *rli39*, *aspocR*, *pdu*, *cob*, *cbi*	21–24
*Lm* signaling RNAs	prfA-5’UTR	Thermosensor controlling *prfA* expression		25
	Anti0677	Downregulates flagellum biosynthesis via MogR	*mogR*, *fliNPQ*	3,26
	LhrA	Regulates ChiA activity required to control iNOS ativity Modulates host immune response via INF- β induction	*chiA*	27 28
	LhrC1–5	Prevents host immune recognition and facilitates adaptation in heme-rich conditions Protects against cell wall-acting antibiotics	*oppA*, *tcsA*, *lapB*, *lmo2185*, *lmo2186* *lmo0484*	29–31
	Rli60	Modulates gene expression in response to BCAAs	*ilvD*	32,33
	RliB	Plays a role in *Lm* adhesion and invasion		34
	SbrE	Involved in isoleucine biosynthesis control	*ilvA*	35
	Rli22	Promotes survival within the intestinal lumen	*lisRK*	36
	Rli27	Modulates levels of a cell wall surface proteins	*lmo0514*	37
	Rli31	Protects against lysozyme activity	*spoVG*	38,39
	Rli32	Protects against cell envelope stresses Modulates genes involved in tryptophan biosynthesis	*virR*, *lmo1958*, *lm1960, hbp1-2* *lmo1627–33*	28,40
	Rli38	Related with organ colonization	*fur*, *lmo0460*, *lmo2752*	3,41
	Rli50	Promotes macrophage proliferation and intracellular survival		28,42
*Lm* signaling via TCSs	β-lactams, lysozyme, ethanol Antimicrobial agents	Sensed by CesK	*lmo2420* *lmo0441*, *lmo2812*	43 44
	β-lactams, acidic conditions, hemoglobulin, ethanol, bile salts	Sensed by LisK	*htrA, lhrC1–5*	31,45–47
	Human defensins, cationic peptides	Sensed by VirS	*dltP*, *mprF*	48
	Gastric conditions	Sensed by PieS	*prsA1-2*	49,50
Host physical signals	Temperature	Triggers *prfA* and PrfA-controlled genes expression Impairs Gmar activity, allowing the expression of *mogR*	*gmaR*, *mogR*	25,51,52
	pH	Activates *Lm* stressosome and σ^β^ to maintain pH homeostasis Controls LLO activity	*rsbR1-2-3-4*, *rsbL-S-T*, *gadD3* *hly*	53–58 59–62
Host nutritional signals	L-cysteine	Required for GSH biosynthesis	*gshF*	63,64
	Cysteine containing peptides	Organic nitrogen source used to induce the activation of PrfA	*opp*	65
	Bile salts	Promotes *Lm* adaptation to the gastrointestinal tract	*bsh*, *brtA, mdrT*, *cadC*	4,66–69
	BCAAs	Modulate virulence gene expression and protein synthesis in a CodY-dependent manner	*codY*, *ilvC*, *purH*, *prfA*, *rli60*	32,70–73
	FFAs	Impair PrfA-dependent gene activation within the gut		74,75
	L-glutamine	Induces *hly*, *plcA*, *plcB* and *actA*	*glnQ*, *glnP*	76,77
	Non-dependent PTS	Carbon sources sensed and metabolized to improve bacteria growth within the host	*hpt*, *prfA*, *glpF*, *inlC*, *hly*, *actA*	78,79
Host chemical signals	Iron	Mitigates ROS-mediated damage in phagosomes Induces InlA, InlB and LAP expression during early infection stages Required for the upregulation of FrvA	*inlA-B*, *lap* *frvA*	80,81 82–84 85,86
	ROS	Trigger regulatory pathways to allow survival within the host	*perR*, *kat*, *fur*, *hemA*, *fri*, *frvA*, *prfA*, *hly*	87–89

### Listeria monocytogenes signaling molecules regulating virulence

Pathogenic bacteria have developed several strategies to survive and persist within host cells and tissues. In *Lm*, most of these strategies are controlled and/or influenced by cell-to-cell communication, a process mediated by molecules produced and secreted by bacteria themselves. These signaling molecules are part of a dialogue between bacteria, and can be either chemical and cell-population density related (e.g quorum-sensing, pheromones) or generated by bacteria at different infection stages (e.g RNAs, second messengers, and glutathione), being both essential to ensure the successful establishment of infection.^90^

## Quorum sensing

Quorum sensing (QS) is a microbial cell-to-cell communication process that relies on the production, secretion, detection, and group-wide accumulation of extracellular signaling molecules named autoinducers. Throughout this process, bacteria share information concerning the population density to accordingly regulate the expression of genes involved in virulence, competition, stress response, and resistance.^91,92^

Pathogenic Gram-positive bacteria typically produce autoinducing peptides (AIPs) as autoinducers, being initially synthesized as precursor peptides (pro-AIPs), modified, and then exported through a specialized protein transport system.^93,94^ AIPs are generally sensed and recognized by a two-component signal transduction system. High cell density communities accumulate higher concentrations of extracellular AIP, which binds to a membrane-bound histidine kinase receptor triggering its own phosphorylation and transferring the phosphate group to a cognate cytoplasmic response-regulator, that ultimately regulate the expression of QS related-genes.^93,95,96^ Moreover, AIPs can be transported back to the bacterial cytoplasm where they are capable to interact with cytoplasmic transcriptional factors, thus modulating their activity and gene expression pattern.^97,98^ The Agr system and its orthologs are the most well-studied circuits of bacterial QS, mainly in *Staphylococcus aureus*, and play a fundamental role in the spatiotemporal expression of virulence mechanisms of Gram-positive human pathogens, including *Enterecoccus faecalis*, *Clostridium perfringens* and *Lm*.^5,99-102^

**Figure 1.**
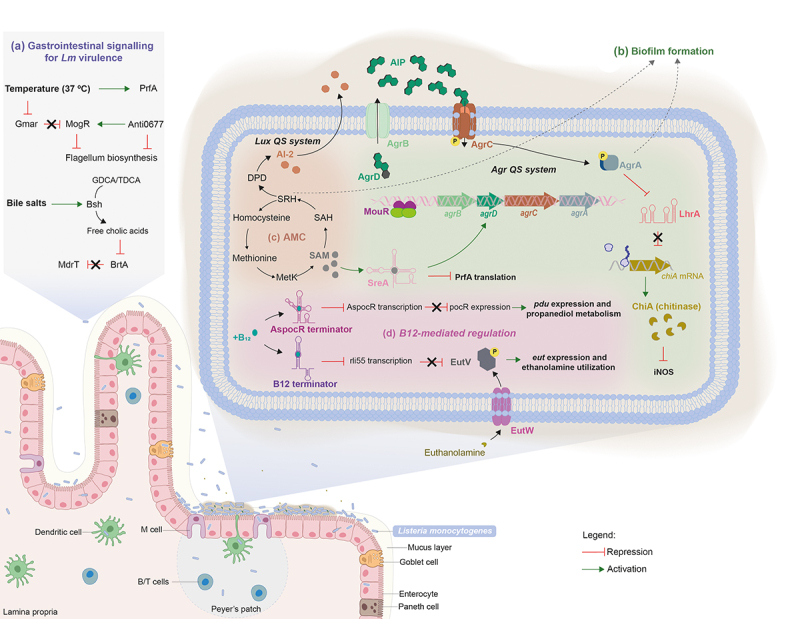
Signalling of *Lm* virulence gene expression during the infection of the gastrointestinal (GI) tract. (a) The temperature within the mammalian host is key for *Lm* to modulate the expression of virulence genes. This thermal cue is sensed both by the *Lm* pleiotropic regulator *prfA*, whose ribosome binding region becomes accessible enabling the translation of downstream mRNA,^25^ and by the anti-repressor GmaR that suffers a conformational shift, allowing the expression of MogR and consequently the repression of genes involved in flagellum biosynthesis. Moreover, the Anti0677 is capable of inducing the transcription of *mogR* by generating a longer *anti0677* transcript, *positioned* on the opposite strand of the flagellum operon coding sequence (*fliN*, *fliP* and *fliQ*).^41^ within the gastrointestinal tract, *Lm* encounters bile salts. It uses Bsh, which is crucial for *Lm* resistance to bile and virulence, to catalyze the hydrolysis of GDCA- and TDCA-conjugated bile salts into free cholic acids, that are exported by the MdrT efflux pump of the bacterium.^66^ Consequently, in the presence of cholic acids, the bile sensor BrtA, which controls *mdrT*, is no longer able to repress *Lm* MdrT.^67^ (b) the ability of *Lm* to internalize intestinal epithelial cells relies, in part, on its capacity to form biofilms at the surface of the intestinal villi, which is dependent on MouR that positively regulates the *agr* operon by binding to its promoter region.^103^ AgrD is processed by AgrB into a mature AIP, that is subsequently secreted to the extracellular medium. The secreted AIP is then recognized by the sensor kinase AgrC present within the neighboring bacteria, triggering a phosphorylation cascade that culminates in the activation of the response regulator AgrA. In turn, AgrA induces the auto-activation circuit. Therefore, the Agr system induces chitinase activity by the down-regulation of the ncRNA *lhrA*, which blocks translation by binding itself to chitinase mRNA (chiA). In turn, chitinase represses the inducible nitric oxide synthase (iNOS), known to be part of the immune response.^27,104^ (c) the activated methyl cycle (AMC) also plays a role in biofilm formation by the generation of the S-ribosylhomocysteine (SHR) by-product, through the processing of S-adenosylmethionine (SAM). Concomitantly, SAM is used to bind and activate SreA riboswitch, that in turn increases the expression of *agrD*.^5,8,10^ Simultaneously, the activation of *Lm* SreA riboswitch by SAM inhibits the *prfA* transcription in the intestine.^3,10^ (d) Additionally, within the GI *Lm* comes across ethanolamine, a metabolite derived from the phospholipids of eukaryotic cell membranes, using it as carbon source to support bacterium growth.^105^ in presence of ethanolamine, the sensor kinase EutW induces the phosphorylation and activation of the anti-terminator EutV, that together with Rli55 repression, upregulate the expression of the ethanolamine operon *eut*. The repression of Rli55 is dependent on a B_12_-riboswitch, located upstream of the *eut* operon, which in the presence of vitamin B_12_ blocks the transcription of Rli55 allowing the expression of *eut* genes. Vitamin B_12_ is also used as cofactor in propanediol metabolism: in the presence of propanediol, B_12_ riboswitch ends prematurely the transcription of AsPocR, allowing the transcription of *pocR*, which is important to activate the *pdu* and *cob* operons, respectively involved in propanediol utilization and B_12_ biosynthesis respectively.^106^

*Lm* QS systems have been associated with bacterial communication and survival providing competitive advantages in adhesion to surfaces, biofilm formation, invasion of mammalian cells, mouse infection, and changes in gene expression (Figure 1).^5,99,107,108^ Two QS systems, Lux, and Agr, widespread among different bacterial species, were already established for *Lm*. The Lux system (Figure 1C) involves the conversion of S-adenosylhomocysteine (SAH) into S-ribosylhomocysteine (SRH) that is further processed to give rise to both homocysteine and 4,5-dihydroxy-2,3-pentanedione (DPD). This latter reaction is achieved by two *Lm* enzymes, Pfs (S-adenosylhomocysteine nucleosidase) and LuxS (S-ribosylhomocysteinase). Thereon, DPD rearranges into a furanone derivative called autoinducer AI-2, which is then released to the extracellular media.^6,12^ AI-2 was found to be important only for the detoxification of SAH, while its precursor SRH is important for biofilm formation.^12^ Importantly, both SAH and SRH are by-products generated in the activated methyl cycle (AMC) from S-adenosylmethionine (SAM) processing. SAM is an intermediate metabolite generated from methionine in the MetK synthase cascade pathway, and is recognized as a sentinel metabolite capable of bind to 5’ UTRs riboswitches controlling the transcription of their downstream genes, mainly involved in the biosynthesis, transport, and utilization of amino acids, oligopeptides and SAM itself: between them, the adenosylmethionine synthetase (*metK*) and the methionine (*lmo2417*) and cysteine (*lm0135*) transporters.^6-9^

The *Lm* Agr QS is encoded by the *agr* locus *agrBDCA* (Figure 1B). *agrD* encodes a ribosomal peptide precursor that is proteolytic processed into a mature AIP by the integral membrane-bound peptidase AgrB.^13^ At this point, in *Staphylococcus aureus*, it is described the formation of an intermediate enzyme-bound thiolactone, which is then transported across the membrane.^109^ However, in *Lm* there is no evidence about the formation of such intermediate. On the other hand, a recent study presented strong evidences that the native *Lm* AIP might be an homodetic pentamer peptide arising from thiolactone.^110^ Once the processed AIP is out of the cell, it binds the sensor membrane-bound histidine kinase AgrC, triggering the phosphorylation of the response regulator AgrA, together acting as a two-component system for downstream gene regulation.^13^ The *Lm* Agr QS system was found to be positively regulated by MouR, a transcriptional regulator belonging to the VanR class of the GntR superfamily of regulatory proteins.^103^ The GntR family consists of helix-turn-helix transcriptional regulators that control several metabolic and biological processes in bacteria. These proteins share a conserved DNA-binding domain at the N-terminal, allowing them to modulate gene expression in response to signals, and an effector binding/oligomerization domain at the C-terminal The diversity of the C-terminal domain is used to divide GntR proteins in different sub-classes, where the proteins composed by six α-helices in the C-terminal are allocated to the VanR class.^111–113^ MouR binds directly to the *agr* promoter region, modulating both chitinase activity and biofilm formation, thus favoring *Lm* virulence.^103^

Previous findings have demonstrated a cooperation between the Agr QS and the SAM signal in the regulation of biofilm formation. The generation of SAM induces the expression of the *agr* locus, with *agrD* appearing as the most expressed gene. In contrast, the absence of the Agr QS system significantly impairs SAM-enhanced biofilm formation, but does not affect the expression of the genes involved in SAM biosynthesis.^8^

Recent efforts have been done to modulate QS and virulence outcome with the goal of designing novel antimicrobial therapeutics. Chemical antagonists able to diminish *Lm* Agr activity and thus leading to the reduction of biofilm formation by over 90% were recently discovered.^114^ Moreover, *in silico* analysis revealed different proteins secreted by lactic acid bacteria working as a promising anti-QS strategy, by inhibiting the translocation of cyclic AIP via AgrB and its recognition by the AgrC active site.^115^

Of note, some *Lm* strains like the well-studied laboratory reference strain 10403S harbor mutation in *agr* genes, being still virulent in mouse infection models.^116^

## Peptide-pheromone pPplA

In addition to the Agr system discussed above, an additional peptide-signaling system has been associated with *Lm* virulence. *Lm* displays a small peptide pheromone, pPplA, that is used to signal its presence within the host cell vacuole, facilitating its vacuolar escape possibly by promoting PrfA activation. pPplA is generated via the proteolytic processing of the PplA lipoprotein secretion signal peptide, been then secreted through the general secretory pathway. When confined into the vacuole, *Lm* is able to sense and import previously produced and accumulated pPlpA, thus initiating a signaling cascade that favors vacuolar disruption and bacterial escape to the host cytosol. This pheromone signaling appears to be linked to PrfA activation.^14^

## RNA-mediated regulation

RNAs have emerged as prime regulators of adaptive response and pathogenesis. This type of regulation is quite present in pathogenic bacteria and provides new information not only about how bacteria can regulate their virulence gene expression but also about their survival associated-phenotypes.^117-119^ RNA molecules can regulate stress response, metabolism and virulence post-transcriptionally, both through translation and stability of mRNAs encoding genes or interacting with specific proteins.^118,120^ Importantly, the RNA-mediated regulation presents an advantage for the bacteria throughout infection, since it provides quick responses with minimal consumption of energy.^121^ Non-coding RNAs (ncRNAs) are functional RNA molecules not translated into a protein. They are crucial gene regulatory elements for *Lm*, both within or outside the host, and were found to play essential roles in many biological processes including signaling, RNA processing, gene regulation and protein synthesis, which condition virulence and host-immune response.^3^ Transcriptomics studies of *Lm* have already identified 46 cis-acting riboswitches, 104 antisense RNAs and 154 putative small trans-acting RNAs.^122^ In this section, we will focus on the most relevant categories of *Lm* ncRNAs governing virulence.

The first category of *Lm* ncRNAs comprises the regulatory elements, usually located in the 5’-untranslated regions (UTRs), and capable to control the expression of virulence genes at the post-transcriptional level.^123^ Their secondary structure generally suffers a conformational change in response to cellular and/or environmental signals including temperature (thermosensors) or ligand binding (riboswitches).^124^ The thermosensors regulation depends on temperature to generate an alternative conformation in which the ribosome-binding site is hidden by the thermosensing stretch of the RNA element, thus modulating protein translation.^125,126^ The most prominent example of this type of regulation in *Lm*, that signals its presence in mammalian hosts allowing the activation of most virulence mechanisms, is the 5’-UTR of *prfA*, the major transcriptional activator of *Lm* adhesins, internalins, phagosome-escaping factors and immune modulating factors.^127–130^ At low temperatures (<30°C), the *prfA*-UTR forms a secondary structure that masks the ribosomal region of *prfA*, avoiding the ribosome binding and consequently inhibiting the translation of PrfA and the expression of PrfA-regulated virulence genes. Temperature rise (>37°C) induces a structural change in the *prfA* 5’-UTR, allowing ribosome access, the translation of *prfA* and the expression of PrfA-dependent genes.^130^ Nonetheless, this thermocontrol appears to act in parallel with other PrfA-activating mechanisms since the temperature increase to 37°C *per se* is not sufficient to induce PrfA-dependent gene expression.^131^ In that regard, the regulation of PrfA is also dependent on two SAM riboswitches, SreA and SreB. Interestingly, these riboswitches, due to their high complementary with *prfA*-5’UTR, may function as *trans*-ncRNAs, inhibiting the transcription of *prfA* mRNA.^10^ Likewise, the absence of SreA decreases the amount of AgrD,^8,10^ which is crucial for invasion of intestinal Caco2-epithelial cells and biofilm formation.^5^ SAM riboswitches being usually activated by the binding of their effector molecule S-adenosylmethionine, allow the formation of a termination structure that ends up gene transcription and inhibits the synthesis of downstream mRNA.^9,11^ However, the SreA-*prfA*-5’UTR interaction is possible only at temperatures permissive for infection (i.e., 37°C), and does not necessarily require the presence of SAM.^10^ Altogether, these findings pointed for a role of SAM riboswitches in *Lm* virulence, downregulating *prfA* expression in the intestine, where it seems to be less critical than in the blood, keeping active other regulatory pathways such as the Agr system.^3,10^ Recently, a chimeric antisense oligonucleotide (ASO) was designed as a novel antibacterial agent targeting SAM riboswitch.^132^

Another regulation dependent on a riboswitch (Figure 1D) concerns the ncRNA Rli55, nearby the *eut* operon involved in the consumption of ethanolamine, an abundant molecule in the human intestine that contributes to *Lm* mouse infection. Vitamin B_12_, also named cobalamin, binds to the B_12_ riboswitch that controls Rli55 expression by ending prematurely its transcription.^21^ Rli55 functions by binding and sequestering the two-component response regulator EutV. Defects in ethanolamine utilization, or in its regulation by Rli55, significantly attenuate *Lm* virulence in mice.^3,21^ The availability of vitamin B_12_ also regulates the Rli39 riboswitch to control the transcription of AspocR, an antisense RNA. Truncated transcripts of *aspocR* arise whenever vitamin B_12_ binds to the riboswitch; contrarily, a full-length transcript is generated in the absence of vitamin B_12_, inhibiting *pocR* mRNA translation, through an antisense mechanism. In turn, PocR up-regulate the expression of the *pdu* genes, which are essential for the propanediol metabolism involved in the pathogenesis of many intestinal pathogens.^22^ PocR was also proposed to control the expression of *Lm cob* and *cbi* genes, that are involved in vitamin B_12_ *de novo* biosynthesis, in an attempt to maintain the production of vitamin B_12_ derived cofactors.^23,24^

The second category of *Lm* ncRNAs comprises the antisense RNAs (asRNAs), including long antisense RNAs (lasRNAs), that act as major negative regulators of gene expression and are encoded on the opposite strand in relation with the genes they regulate.^123^ These type of ncRNAs have full complementary sequence with the mRNA, providing not only strong and specific bindings, but also more efficient action, thus rapidly conditioning mRNA stability and translational activity.^120,133^ Anti0677 is a lasRNA, named excludon, crucial for *Lm* infection, that negatively regulates the flagellum biosynthesis, in particular through the control of the MogR transcriptional repressor of flagellum genes. *mogR* transcription occurs from two alternative promoters: the P1 promoter is located upstream the *mogR* start codon and produces a shorter and constitutive transcript, whereas the P2 promoter is σ^B^-dependent and generates a longer *anti0677* transcript that contains *mogR* and originates on the opposite strand to the coding sequence of the *fliNPQ* flagellum operon.^3^ The expression of *anti0677* leads to higher transcription of *mogR* and decreased levels of *fliNPQ* transcripts, in turn inducing a strong repression of flagellum genes and reduced *Lm* motility.^26^

The third category of ncRNAs involved in *Lm* virulence embraces the *trans* regulatory RNAs (*trans*-ncRNAs), which are encoded *in trans* in relation to the genes they regulate, and are capable both to regulate multiple mRNAs playing with their translation and stability, and to interact with proteins.^123^ *Trans*-ncRNAs can act either as antisense or sense RNAs, and this regulation may happen by two different ways: either the *trans*-ncRNA and its target mRNA are encoded on the same DNA locus, but transcribed in opposite directions leading to the formation of extended base-pairings or, the *trans*-ncRNA is encoded in a different location of the target mRNA, generating a weaker, short and imperfect complementarity with the mRNA, but allowing to control more than one target mRNA.^133-136^ *Lm* displays several *trans*-ncRNA involved in its pathogenicity, namely LhrA, LhrC1–5, Rli33–1 (LhrC6), Rli22 (LhrC7), RliB, Rli47 (SbrE), Rli27, Rli31, Rli32, Rli38 and Rli50.^3^

LhrA is a *trans*-ncRNA with a role in the transition from exponential to stationary phase of *Lm* growth, whose stability is dependent on the presence of the Hfq chaperone^137^ Transcriptomic studies clearly showed that *lhrA* inactivation alters the expression levels of more than 300 *Lm* genes, including the *chiA* gene that encodes *Lm* chitinase A important for *Lm* pathogenesis in mice, possibly by targeting host proteins involved in immune signaling.^27,138,139^ *Lm* LhrA may modulate host immune response throughout infection by inducting interferon-beta (IFN-β) production.^28^ In addition, the QS Agr system promotes chitinase activity through negative regulation of this short ncRNA *lhrA*, which itself binds to chitinase mRNA (*chiA*) blocking ribosome access and translation.^104^ In turn, ChiA secretion by *Lm* downregulates the inducible nitric oxide synthase (iNOS) activity in infected mice, enhancing bacterial survival.^27^ MouR, a transcriptional activator of the *Lm* Agr system, was also shown to repress the expression of *lhrA*.^103^

*trans-*ncRNAs belonging to the LhrC family, including LhrC1–5, Rli33–1 and Rli22, were shown to be induced when *Lm* reaches the human bloodstream.^3^ Such is the case of LhrC1–5, which is upregulated when bacteria are exposed to the heme group of the human hemoglobulin and positively activated by the response regulator LisR, part of the two-component system LisRK.^29,30^ Moreover, LhrCs were shown to repress, at the post-transcriptional level, different *Lm* virulence-associated genes (*oppA*, *tcsA* and *lapB)*, acting by base pairing to the Shine-Dalgarno (SD) region, thus decreasing mRNA levels or inhibiting translation. This regulation by LhrC1–5 is a competitive advantage throughout human blood infection under heme-rich conditions. The downregulation of these surface exposed proteins prevents immune recognition and enhances bacterial survival within the host.^29–31^ Additionally, in the presence of the β-lactam antibiotic cefuroxime, LhrC1–5 downregulates the expression of a heme oxygenase-like protein Lmo0484, by base paring to the ribosomal binding site and consequently inhibiting its translation. This repression was found to protect *Lm* against this cell wall-acting antibiotic.^29,30^ Other examples of ncRNAs belonging to the LhrC family and playing a role in *Lm* virulence are Rli33–1 and Rli22. The lack of *rli33–1* has been found to significantly impair *Lm* survival within macrophages and virulence both in murine and insect models of infection, suggesting a critical role for this ncRNA to favor *Lm* persistence within host cells.^42^ The expression of *rli22* was found to be upregulated in bacteria inside the intestinal lumen of mice or upon bacteria exposure to cell envelope stress in a LisRK two-component system manner.^3,36^

Furthermore, there is a significant correlation between the ncRNA RliB and *Lm* virulence, as demonstrated by the upregulation of this ncRNA in key infection sites such as the mouse intestinal lumen and the blood.^3^ RliB plays a role in the adhesion and invasion ability of *Lm* but mechanisms underlying its upregulation and functional role during infection require further research.^34^

Rli47, also named SbrE, is a σ-dependent ncRNA highly induced in the intestinal lumen and in macrophages.^3,42^ *In silico* and *in vivo* analysis of SbrE structure reveal its role in binding the SD region of *ilvA* mRNA, encoding a threonine deaminase involved in the biosynthesis of branched-chain amino acids.^35^ This interaction occludes the *ilvA* ribosome binding site, thus repressing its translation. Consequently, the threonine deaminase enzymatic activity is repressed and the initial step of the L-isoleucine biosynthetic pathway blocked. These observations highlight the role of SbrE in the isoleucine biosynthesis repression under harsh conditions, inhibiting *Lm* growth and impacting virulence.^35^

Rli27, which is upregulated in the mouse intestinal lumen and in blood,^3^ was implicated in the post-transcriptional regulation of *lmo0514* during the infection process.^37^ Lmo0514 is an internalin-like protein covalently linked to the bacteria cell wall by a LPXTG motif. It is upregulated during *Lm* colonization of eukaryotic cells in a PrfA and σ^B^-independent manner,^140^ and is required for bacterial survival in plasma, having a role in virulence at early infection stages.^141^ Rli27 interacts with *lmo0514* long 5’-UTR, inducing its translation and modulating the cell wall surface protein levels during intracellular infection. Within the eukaryotic niche, this regulation depends on the activity of two promoters of the target gene, generating a short and a long 5′-UTR transcripts, whose relative abundance varies from extra- to intra-cellular bacteria. Importantly, the sRNA binding site is only present in intracellular bacteria, once they are enriched with the long 5’-UTR version.^37^

The transcription of both *rli31* and *rli32* ncRNAs increases significantly during *Lm* infection of macrophages.^42^ These ncRNAs were also found to play a role in *Lm* virulence.^28,38,39^ The absence of *rli31* renders *Lm* more susceptible to the action of lysozyme, leading to a significant attenuation of *Lm* virulence upon intravenous mice infection. These findings highlight the critical role of Rli31 in protecting *Lm* from the enzymatic activity of lysozyme present in the bloodstream.^38^ In addition, *in vitro* experiments confirmed that Rli31 is capable to bind to the 5’-UTR of *spoVG* mRNA, a regulator implied in lysozyme resistance, motility and virulence. However, the absence of *rli31* does not impact *spoVG* mRNA or protein levels. Curiously, the absence of both Rli31 and SpoVG induces a contrary effect on the regulation of lysozyme resistance and virulence, probably implying an antagonistic regulatory relationship between Rli31 and SpoVG, that allows bacteria to fine-tune their growth in different environments.^39^ Rli32, which also binds to SpoVG protein, was found to be secreted from bacteria to the host cytosol upon infection, triggering the expression of β-interferon.^28,39^ Additionally, the transcription of *rli32* was found to be dependent on VirR, a transcriptional regulator of *Lm* virulence, suggesting that Rli32 plays a role in virulence and/or resistance to cell envelope stress underscoring its importance in bacterial adaptation and survival strategies.^40^ The absence of *rli32* leads to a decrease in *Lm* ability to survive within macrophages and promotes the downregulation of the ncRNA Rli60 and genes responsible for the tryptophan biosynthesis (*lmo1627–33*). Conversely, overexpression of *rli32* favors *Lm* intracellular growth and renders the bacteria more susceptible to the action of cefuroxime. The Rli32 overexpression induces *Lm* resistance to H_2_O_2_-induced stress and also increases the expression of the ncRNAs LhrC1–4, genes encoding the ferrichrome ABC transport (*lmo1958* and *lmo1960*), and the heme-binding proteins Hbp1 and Hbp2.^28^ These intricate findings highpoint the multifaceted roles of Rli32 in *Lm*, impacting various cellular processes, including host interactions, stress responses, and antibiotic susceptibility.

Rli38 was shown to be associated with *Lm* colonization of mouse organs.^3,41^ This *trans*-ncRNA is capable to target mRNAs of three different genes, including the global iron uptake regulator Fur, related with bacterial adaptation in blood, the membrane associated lipoprotein Lmo0460 that belongs to the internalin family, and the ABC transporter ATP-binding protein Lmo2752. During an oral infection of mice, the absence of Rli38 attenuates *Lm* virulence.^3^

Another example of a *trans-*ncRNAs impacting *Lm* virulence is Rli50 which exhibits higher transcription levels intracellularly. The absence of *rli50* not only impairs the bacterium capacity to proliferate within macrophages but also results in virulence attenuation in both insects and mice.^28,42^ These findings highlight the role of Rli50 to promote *Lm* intracellular survival, pathogenicity and an adequate host immune response.

The last category of ncRNAs is composed by the *cis*-regulatory RNAs (*cis*-ncRNAs). Unlike *trans*-ncRNAs which can act on genes located at different genomic locus, *cis*-ncRNAs mostly exert their regulatory effects on the same genomic region where they are transcribed from. These ncRNAs can adopt two alternative RNA structures, leading either to transcription termination or transcription antitermination of downstream genes.^118,120^ Such is the case of *rli60*, a *cis*-ncRNA located upstream of an operon responsible for the biosynthesis of branched-chain amino acids (*ilvD*), that is transcribed whenever levels of these amino acids are limited.^32^ Rli60 was implicated in *Lm* virulence in a mouse model, especially due to the capability of *Lm* to invade and proliferate within the liver, spleen and macrophages.^33^ Interestingly, the absence of *rli60* was correlated with increased adhesiveness to macrophages and decreased ability to form biofilms.^33,142^ It was also suggested that *Lm* Rli60 is able to sense the available branched-chain amino acids, that are essential for its optimal growth, downregulating downstream genes accordingly. This regulation favors bacterial pathogenicity by ensuring the proper levels of internal branched-chain amino acids, which are *per se* a signal for the CodY regulator to bind *prfA*, thus allowing the expression of a number of major *Lm* virulence factors.^32^

## Second messengers

**Figure 2.**
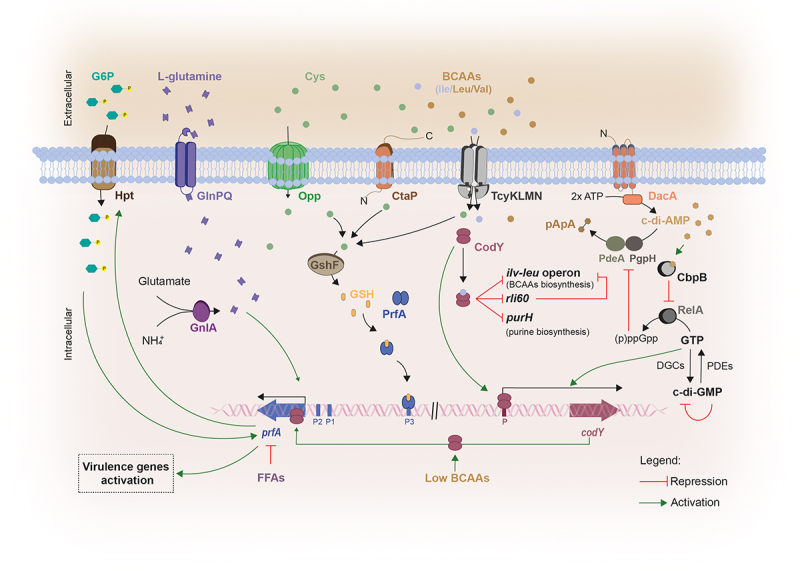
The interplay between host nutritional signals and *Lm* signaling molecules in virulence. Within the host, *Lm* imports G6P via the PrfA-regulated permease Hpt, to support its intracellular growth.^79^ Additionally, *Lm* uses host L-glutamine whose uptake is dependent on the ABC transporter GlnPQ, to further induce the expression of PrfA-controlled virulence genes. *Lm* is also capable to synthesize L-glutamine, even not being enough to induce virulence genes, in the presence of ammonia and glutamate by using the glutamate synthase GnlA.^77^ the expression of *prfA* is also dependent on glutathione (GSH), which is synthesized from the host cytosol imported L-cysteine, via the glutathione synthase GshF. L-cysteine uptake is mediated by three different transport systems: the substrate-binding CtaP, the Opp transporter and the ABC transporter TcyKLMN. Once synthesized, GSH binds PrfA, inducing a conformational change that enables its binding to the the PrfA box within the promoter regions of PrfA-regulated genes, thereby activating their expression.^106^ Importantly, *prfA*-transcription is enhanced in response to the low availability of branched-chain amino-acids (BCAAs) through the binding of CodY to its coding sequence.^73^ Under high concentrations of BCAAs, CodY binds to isoleucine repressing the expression of both *ilv-leu* operon and *purH* gene, involved in BCAA and purine biosynthesis respectively, and also *rli60*. In contrast, isoleucine-unbound CodY can bind to DNA, allowing its own expression and inducing the expression of *prfA* and consequently PrfA-regulated genes. When challenged with low BCAA levels *rli60* is transcribed forming a ribosome-mediated attenuator that restricts the expression of genes belonging to the *ilv-leu* operon, in turn increasing *Lm* virulence through CodY expression.^32^ CodY-mediated regulation is also dependent on c-di-AMP levels. c-di-AMP is synthesized from two ATP molecules via DacA and degraded by PDEs, PdeA and PgpH, giving rise to pApA. High c-di-AMP concentrations induce the up-regulation of CodY regulon, via CbpB-mediated inhibition of RelA activity.^16^ This inhibition ensures the maintenance of GTP levels derived from the degradation of c-di-GMP, that induce the expression of CodY.^145^

Interpreting extracellular information also requires signal transduction across the bacterial cell membrane. Therefore, intracellular small molecules such cyclic dinucleotides were found to participate in gene transduction through the transmission of signals/stimulus to effector molecules.^143,144^ Importantly, second messenger systems can assimilate several sensory inputs modulating the microorganism response output.^143^ *Lm* uses two major types of intracellular second messengers to modulate its virulence: the cyclic di-adenosine monophosphate (c-di-AMP) and the cyclic di-guanosine monophosphate (c-di-GMP) (Figure 2).

The synthesis of c-di-AMP is required for full *Lm* viability both in *in vitro* conditions and within the host.^15,146^ This process is catalyzed by a diadenylate cyclase, DacA, from two molecules of ATP and degraded by two phosphodiesterases (PDEs), PdeA and PgpH.^147^ Mutants lacking the *dacA* gene have been found to exhibit reduced infection capability in a mouse model, increased susceptibility to cell wall antibiotics and inability to growth in rich media without salt supplementation.^146,148,149^ Moreover, a decrease in c-di-AMPs levels upon DacA depletion or PDEs overexpression results in decreased bacterial replication into primary bone marrow-derived macrophages.^146^ DacA has been associated with the induction of IFN-β expression during infection, triggering the innate immune response thereby leading to the activation of host cellular defense mechanisms to control infection.^150^ The c-di-AMP binding protein CbpB acts as a c-di-AMP sensor and activates the synthesis of the stringent response regulator (p)ppGpp (guanosine penta-phosphate) by binging to the bifunctional (p)ppGpp synthetase/hydrolase RelA. High c-di-AMP concentrations preventes RelA activation by binding and sequestering CbpB. (p)ppGpp is also a second messenger that binds and inhibits c-di-AMP phosphodiesterases, resulting in an increase in c-di-AMP. CodY is a pleiotropic transcriptional regulator that represses nutrient uptake and amino acid biosynthesis genes in response to the levels of GTP and branched-chain amino acids. c-di-AMP appears essential to regulate (p)ppGpp levels because accumulated (p)ppGpp altered GTP concentrations, thereby inactivating CodY.^15,16^ c-di-AMP was also shown to bind and regulate the enzyme pyruvate carboxylase allowing bacterial proliferation and subsistence within the host.^17^

c-di-GMP synthesis is accomplished by diguanylate cyclases (DGCs) from two GTP molecules. Beyond being degraded by PDEs, c-di-GMP is able to inhibit its own expression by binding itself to its inhibitory site in order to avoid an excessive GTP consumption.^151-153^ Low c-di-GMP levels were found to be required for bacteria to induce an acute infection, while high levels of c-di-GMP are necessary for a long-term host colonization (chronic infections).^154^ By binding to effector molecules, c-di-GMP regulates several cellular processes including virulence, QS systems, cell morphology, differentiation, cell–cell communication, exopolysaccharide matrix production, flagellum biosynthesis and pilus assembly.^155-158^ Specifically, for its activity at the gene expression level, c-di-GMP interacts with RNAs such as riboswitches and DNA-binding proteins.^159^ In *Lm*, it was demonstrated that high c-di-GMP levels play a negative role on virulence, by inducing the synthesis of an exopolysaccharide that was associated with motility inhibition, cell aggregation and enhancement of tolerance to disinfectants and desiccation. Moreover, high levels of c-di-GMP were found to alter the expression of internalin genes by downregulating the entire virulence regulon, including the master regulator PrfA, thus diminishing not only enterocyte invasiveness *in vitro* but also the bacterial load in the liver upon oral mice infection.^145,158^ It was proposed that elevated c-di-GMP levels induce lower GTP levels, decreasing the activity of CodY, the coordinator of metabolism and virulence, in turn suppressing *Lm* virulence *via* decreased PrfA expression.^145^

Additionally, the degradation of both c-di-AMP and c-di-GMP can result on the formation of two linear dinucleotides, pApA and pGpG, respectively, that are important products of the RNA metabolism.^151,160,161^ Both molecules can also be hydrolyzed by the oligoribonucleotidase NrnA, generating the mononucleotides AMP and GMP, respectively. Concomitantly, the *nrnA* gene was also found to be up-regulated during *Lm* biofilms formation.^162^

## Two-component systems

**Figure 3.**
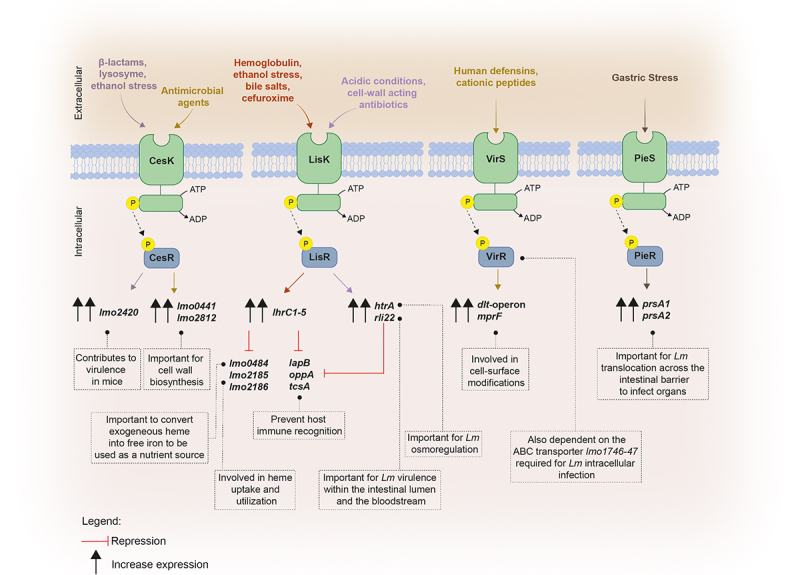
Schematic representation of the two-component transduction systems governing virulence gene expression in *Lm*.

Beyond the signaling molecules previously discussed, *Lm* employs other regulatory mechanisms that contribute to establish infection. Among them, two-component transduction systems (TCSs) stand as paramount players enabling bacteria adaptation to the conditions encounter within the host. These bacterial systems are typically composed by a transmembrane sensor histidine kinase and a cytoplasmic response regulator, both working together to sense and process external stimuli including nutrients, metal ions, host-derived metabolites and gut specific conditions. External cues usually trigger the activation and autophosphorylation of the histidine kinase, and the phosphoryl group is transferred to the cognate response regulator, which in turn is activated to work as a transcription factor that regulates gene expression.^163,164^ So far, among the 14 TCSs of *Lm*, four have been related to its pathogenicity: CesRK, LisRK, ViRS, and PieRS (Figure 3).

CesRK, which is composed by the cephalosporin sensitivity response regulator CesR and its sensor histidine kinase CesK, is implicated in sensing and responding to changes in the cell wall integrity and in the expression of cell envelope-related genes.^43,44^ In the absence of *cesR, Lm* becomes less virulent in mice upon intragastric infection, but not after intraperitoneal inoculation, thus suggesting a prime role for CesRK TCS in *Lm* pathogenicity.^43^ Furthermore, CesRK is capable to sense not only the presence of β-lactam antibiotics typically used to treat *Lm* infections, but also ethanol and lysozyme, further inducing the transcription of the downstream small open reading frame *orf2420* (*lmo2420*), also important for bacterial pathogenesis.^43^ *Lm* CesK perceives cell wall damages caused by antimicrobial agents, generating a faster response by the bacteria that activate a cascade of events that culminate in the upregulation of key genes involved in cell wall biosynthesis, such as *lmo0441* and *lmo2812*.^44^

LisRK was found to be implicated in the transcription regulation of the *htrA* gene, that encodes a HtrA-like serine protease involved in *Lm* osmoregulation and pathogenesis,^45^ and conferring protection against some β-lactams antibiotics such ampicillin.^46,47^ It was suggested that *Lm* LisRK is able to sense perturbations in the surrounding environment, such as the acidic conditions or the presence of cell wall-acting antibiotics.^45^ LisRK also plays a role in the invasion capacity of the pathogen, mutant bacteria becoming significantly less virulence upon mice infection.^165,166^ Additionally, this TCS together with the *trans-*ncRNAs LhrC1–5, are important for intracellular replication of *Lm* into macrophage-like cells.^31^

The VirRS system, which composes another TCS, acts as a positive transcriptional regulator of genes mainly involved in cell surface modifications, such as the *dlt*-operon and *mprF*, which are crucial for bacteria to fight human defensins and cationic peptides.^48^ The absence of the *virR* gene is associated with the impairment of *Lm* cell invasion and infection capacity in mice. Interestingly, VirR activation was shown to be not totally dependent on its cognate kinase, since it also depends of an ABC transporter encoded by the genes *lmo1746–47* shown to be required for optimal intracellular infection and virulence.^40,48^ Contrarily to others TCS where both genes encoding the sensor and the response regulator are contiguous on the chromosome, *virR* and *virS* are not adjacent but separated by three genes. Likewise, VirS appears to interact with other response regulators, regulating genes that are not affected by the absence of *virR*, thus suggesting a non-exclusive interaction between VirS and VirR.^48^

The TCS PieRS plays a role in promoting *Lm* translocation across the intestinal barrier by regulating the secretion of two chaperones, PrsA1 and PrsA2, both found to be associated to the membrane and secreted in the extracellular medium.^49^ PrsA2 plays a significant role in hemolytic and phospholipase activity, being important for *Lm* cell-to-cell spread and full virulence following both oral and intravenous mice infection,^49,50,167–170^ while PrsA1 is required for *Lm* traffic from the intestine to the liver.^49^ It was proposed that when *Lm* is faced with harsh conditions, such the gastric stress found in the gastrointestinal tract, PieS, the sensor kinase, triggers the activation of the response regulator PieR, leading to the activation of both *prsA1* and *prsA2*, thus enabling bacterial survival and crossing the intestinal barrier to infect target organs.^49^

## Glutathione

In an attempt to mediate *Lm* transition to a pathogenic state, it can use the tripeptide glutathione (GSH) as a metabolic signaling molecule modulating its virulence (Figure 2). This process relies on the allosteric binding of GSH to the major *Lm* transcriptional regulator PrfA, inducing a conformational change on PrfA and therefore its binding to DNA. Consequently, virulence genes regulated by PrfA are expressed, promoting infection.^18-20^ Within bacteria, GSH levels are very limited.^63^ Even so, *Lm* is able to synthesize GSH by making use of its glutathione synthase (*gshF*).^18,64^ GshF is a bifunctional enzyme able to synthesize a GSH intermediate, γ-glutamylcysteine, by binding the γ-carboxyl group of L-glutamate to L-cysteine, previously imported from the surrounding environment since *Lm* is not able to synthesized it. Subsequently, GshF mediates the fusion of this intermediate product with glycine to yield GSH (also known as L-γ-glutamyl-L-cysteinylglycine).^64,171^ On the other hand, inside mammalian cells *Lm* encounters abundant concentrations of GSH being able to import and use it as a signaling molecule to regulate the expression of virulence genes. Curiously, the host-synthesized GSH cannot completely replace the GSH produced by the bacteria itself to activate PrfA and PrfA-dependent genes. Even though *Lm* strain defective for *gshF* do not suffer a general loss of fitness, it exhibits a decrease in the expression levels of some virulence factors, such as the actin assembly-inducing protein ActA required for *Lm* intracellular motility, suggesting that extracellular GSH could function as a signal for bacteria into the host intracellular niche.^18,172^ Importantly, L-cysteine presents itself an amino acid rate-limiting in the synthesis of GSH, directly affecting the expression of *prfA* and PrfA-controlled genes, hence limiting *Lm* virulence.^171,173^ Three different transport systems were already described as being involved in the acquisition of cysteine for GSH biosynthesis: (i) CtaP, a substrate-binding component of a transport system for L-cysteine (ii) the OppABCDF transporter, capable to import exogenous cysteine-containing peptides and (iii) the ABC transporter TcyKLMN capable to collect both cysteine and its reduced form cystine from the extracellular medium.^63,65,174^ Even being all implicated in the bacteria virulence, only the TcyKLMN system appears to be linked to *Lm* growth within the host cytosol by supplying L-cysteine for GSH synthesis.^63^ The TcyKLMN system is controlled by three metabolic regulators CodY, CymR and CysK. When faced with limited availability of branched-chain amino acids, *Lm* up-regulates the TcyKLMN system to uptake cysteine from the extracellular medium, thereby promoting GSH biosynthesis and consequently PrfA activation.^63^ A *gshF* mutant was shown to have a defect in growth *in vitro*, in the presence of diamide and copper ions stress. Moreover, it was shown that *gshF* is required for efficient invasion and proliferation both in macrophages and mice organs. GshF plays a role in *Lm* response to oxidative stress, inhibiting the transcription of the *lmo1997-lmo2004* operon, that encodes a phosphoenolpyruvate-carbohydrate phosphotransferase system, being *lmo2002* the most critical gene to resist to oxidative stress.^175^

Additionally, *Lm* is able to use inorganic sulfur thiosulfate and H_2_S as L-cysteine sources, supporting its growth and virulence. This process occurs via the *Lm* two-step biosynthetic pathway of L-cysteine, that involves the conversion of L-serine into L-cysteine by the CysE/CysK enzymes.^176^ Despite these observations, the mechanism by which *Lm* imports thiosulfate remains unclear.

Furthermore, the toxic by-product of cellular metabolism methylglyoxal, produced by both the host and the bacteria, was recently found to be associated with PrfA activation since it functions as a host cue for *Lm* to produce GSH. *Lm* displays a GSH-dependent methylglyoxal detoxification system composed by two enzymes, GloA (glyoxalase A) and GloB (glyoxalase B) that mediate the detoxification of methylglyoxal by conjugation with glutathione. During *Lm* growth within activated macrophages, it faces higher levels of methylglyoxal, which consequently triggers the activation of *gshF*, therefore resulting in increased levels of bacteria GSH and PrfA activation.^177^

### Host nutritional and physical signals triggering Listeria monocytogenes virulence

Environmental cues signaling the colonization of the bacteria into the host are implicated in the regulation of bacterial virulence factors. Among them, the host temperature, pH, iron availability, bile salts, nutrients and the oxidative stress play key roles on how *Lm* fine-tunes gene expression and protein translation.^178^ This section will focus on the impact of these external signals in *Lm* pathogenesis.

## Temperature

Temperature increase is sensed by bacteria when they shift from the external environment to mammalian hosts. Many bacteria are thermally regulated and thus may alter the expression of their virulence genes to promote infection.^179^ Moreover, the temperature may not only influence the DNA topology, RNA structure and metabolism, as it may impact the activity and processing of proteins.^178^

For *Lm*, the temperature increase to 37°C within the host is a signal to activate the expression of the PrfA thermosensor, triggering the expression of a number of major virulence genes (Figure 1A). At temperatures below 37°C, the mRNA 5’-UTR of *prfA* forms a secondary structure which masks the ribosome binding region, repressing the translation of the downstream mRNA. The temperature increase to 37°C makes impossible the formation of this inhibitory RNA structure, inducing the expression of *prfA* and PrfA-controlled virulence genes.^130,180^ Moreover, the temperature rise also triggers a conformational shift in the anti-repressor GmaR, allowing the expression of MogR and consequently, the repression of genes involved in flagellar-based motility (Figure 1A). While flagellar motility is indispensable for bacterial survival in the environment for nutrient acquisition and surface colonization, within the host, flagella is highly immunogenic working as a signal that activates the immune system, and thus detrimental for the virulence.^51,52^

## pH

After entering the human body, *Lm* encounters resistance against colonization imposed either by the harsh conditions of the gastrointestinal tract or by the host immune system.

Acidic environments trigger the activation of the *Lm* stressosome, a stress sensor that induces a general adaptative stress response crucial to confer acid resistance.^53^ *Lm* stressosome is a dynamic signaling multi-protein complex composed by RsbR1 (*lmo0889*) and its paralogues RsbR2 (*lmo0161*), RsbR3 (*lmo1642*), RsbR4 (*lmo1842*) and RsbL (*lmo0799*), the scaffold protein RsbS and the serine – threonine kinase RsbT. Stressosome activation is achieved by RsbR1 and RsbS that sense and transduce the acid stress signals. This sensing is dependent on the phosphorylation of RsbR1 (at residue T209) and RsbS (at residue S56), both mediated by the RsbT kinase. Subsequently, RsbT is release from the stressosome and interacts with the RsbU phosphatase, which consequently promotes the dissociation of the anti-sigma factor RsbW from σ^β^. These events culminate in the upregulation of genes involved in the general stress response and infectivity, thus favoring the host invasion.^53-56^ Moreover, the activation of σ^β^ by the stressosome is also required for the activation of the glutamate decarboxylase enzyme *gadD3*, that converts L-glutamate into γ-aminobutyrate (GABA) while consuming an intracellular proton, and of the arginine (*arcA*) and agmatine (*aguA1*) deiminases, which metabolize L-arginine into citrulline and ammonia to adjust the cytoplasmic pH.^54,181^ During the first process, the consumption of one proton itself is enough to increase the intracellular pH thus attenuating acidic stress.^182^ GadD3 together with GadD1 and GadD2 enzymes and the glutamate/GABA antiporters (GadT1 and T2) form the glutamate decarboxylase (GAD) system which is key to maintain *Lm* pH homeostasis in acidic environments.^57,183,184^ The GAD system comprises two subsystems that differ depending on the location of the required glutamate: the first involves the uptake of extracellular glutamate by antiporters, which undergoes subsequent decarboxylation resulting in the production of GABA. Subsequently, GABA is exporter back by the same antiporter mechanism while more glutamate is being imported; for the second subsystem, *Lm* uses and processes intracellular glutamate to regulate its intracellular pH, in a process dependent on GadD3 and GadD1.^184,185^ GAD is also involved in *Lm* survival under oxidative stress, namely by conferring resistance to hydrogen peroxide.^58^

Macrophages are capable to recognize and phagocytose the bacteria, engulfing them into intracellular vacuoles termed phagosomes. When cells are infected by *Lm*, the phagosome where the bacterium is contained acidifies to a pH around 5.5 to 6. This acidification works as a switch to induce listeriolysin O (LLO) pore-forming activity *per se* mediating the vacuolar disruption and allowing bacteria release from the phagosome and spread.^59-62^ The cytolytic activity of LLO involves the binding and subsequently oligomerization of LLO monomers within the intracellular vacuole membrane leading to the formation of pores and consequently vacuole disruption. Importantly, LLO activity is intricately linked to the pH environment and controlled by an acidic triad present in the transmembrane domain (D3) of LLO that functions as pH sensor. In stark contrast to the acidic environment encountered within the phagosome, inside the host cell cytosol *Lm* faces neutral pH that destabilizes the D3 domain of LLO promoting an inactivation of LLO by the formation of irreversible denatured monomers. This dual-regulation mechanism highlights the sophisticated interplay between pH dynamics and the functional modulation of LLO in the context of *Lm* infection.^61,186,187^ Importantly, the pH-dependency also avoids uncontrolled LLO activity and consequently its insertion into the host cell membranes that would leave the bacteria constantly exposed to extracellular threats.^62^

## Iron

Iron, a vital element for the growth of almost all living cells, is involved in important cellular functions such as the transport and storage of oxygen, and plays a role on the bacteria toxicity and pathogenesis.^188-190^ Although this element is abundant in the environment, the available free iron in the host is restricted. Therefore, the adaptation to shifts between high and low free iron environment is an important signal for bacteria to regulate their gene expression.^189^ *Lm* needs iron for growth and survival during infection namely to establish itself in the gastrointestinal tract.^191^ To acquire iron, *Lm* displays different mechanisms involving the use of iron reductases and ferric citrate uptake systems. Additionally, *Lm* can use siderophore like-uptake systems.^192,193^ Moreover, the host-derived heme found in hemoglobulin and other hemoproteins, also represents an important source for iron and can be used as metabolic cofactor for many bacteria including *Lm*.^82,192^ Into the host cytoplasm, *Lm* may use iron to support its growth and proliferation and to be protected from the ROS-mediated damage. Furthermore, to mitigate ROS-mediated damage in phagosomes, *Lm* may be also able to sequester iron.^80,81^ Some studies have reported *Lm* ability to use the iron available to assess its location and regulate its behavior in term of gene expression, accordingly. In the presence of higher iron concentrations found in the human gut,^194^ the invasins InlA and InlB (both required for the invasion of non-phagocytic cells including intestinal epithelial cells)^195^ are positively regulated, probably to enhance *Lm* internalization into intestinal cells.^83,84^ Contrary, when bacteria are under iron starvation conditions, the expression of both LLO and ActA is increased for *Lm* to escape from the vacuole, reach the host cytosol, and spread to neighboring cells.^83,84,196^ Additionally, when *Lm* encounters an heme rich environment, it induces the upregulation of FrvA, a specific Fe(II) exporter ATPase part of the PerR (a peroxide-inducible stress response regulator) and Fur (a ferric uptake regulator) regulons.^85^ The absence of *frvA* induces a significant attenuation of virulence in mice.^86^

*Lm* is also using heme as a host-derived signaling molecule to control the expression of the *Listeria* adhesion protein (LAP)^82^ a virulence factor that induces intestinal epithelial barrier dysfunction to promote *Lm* translocation.^197^

## Bile Salts

During the initial infection stage, *Lm* encounters bile salts in the gastrointestinal tract using them as signals to ascertain its location and to adapt to the surrounding environment.^67^ Bile salts are synthesized in the liver, using cholesterol as precursor, being then conjugated with glycine or taurine and released into the small intestine from the gallbladder.^198^ About 10% of the *Lm* genome was found to be regulated by bile. Among them, the bile sensor BrtA was found to be induced.^67^ The bile salt hydrolase *bsh* is expressed when *Lm* is in the gastrointestinal tract to promote bile salts resistance catalyzing the hydrolysis of glycodeoxycholate (GDCA) and taurodeoxycholate (TDCA) conjugated bile salts into cholic acids (Figure 1A).^66^ Consequently, BrtA exposure to cholic acids inhibits BrtA binding to the promoter of the multi-drug efflux pump *mdrT*, inducing MdrT expression and allowing the export of cholic acids.^67^ Intriguingly, the overexpression of *mdrT* can be detrimental for *Lm* virulence *in vivo*,^68^ highlighting the importance of *Lm* in controlling the *bsh* expression levels in the course of the infection. In this sense, *Lm* uses the transcriptional repressor CadC to repress *bsh* expression at late infection stages, avoiding the detrimental overexpression of *mdrT* during this step of the infection.^4,69^ The efflux pump MdrT is also capable to transport c-di-AMP,^199^ inducing a type I IFN-β response which enhances the susceptibility to *Lm* infection,^200–202^ reinforcing the importance of *mdrT* regulation by CadC.

## Peptides

Bacterial pathogenicity is significantly influenced by the availability and acquisition of nutrients from the host environment. Different *Lm* auxotrophic mutants for several nutrients including uracil, phenylalanine, glycine, proline or nicotinic acid were found to retain full virulence, suggesting that *Lm* can acquire these nutrients from the host environment. Interestingly, these strains seem to be able to utilize other nutrient sources such as peptides.^203^ Additionally, *Lm* is capable of exploiting exogeneous oligopeptides, an ubiquitous source of organic nitrogen, to sense the environment and control PrfA activity. Indeed, PrfA activation has been shown to be dependent on the presence of cysteine containing peptides (Cys/Cys-peptides) in the host environment, while being antagonized by oligopeptides lacking cysteine, such as leucine.^65,78,204^ While the activation of PrfA requires occupancy of both GSH sites in the PrfA dimer, nonspecific peptide binding (such as leucine dipeptide) to one monomer is enough to change the symmetry of the two helix-turn-helix motifs, impairing DNA binding and consequently, the expression of virulence genes.^65^

## Branched-chain amino acids (BCAAs)

The availability of branched-chain amino acids (BCAAs), including isoleucine, leucine, and valine, constitutes another important metabolic signal sensed by *Lm* (Figure 2). BCAAs represent an important source of nutrients for bacteria and are crucial to remodel their virulence gene expression, protein synthesis and to control bacterial adaptation to amino acid starvation.^70^ The response to BCAAs is dependent of CodY, a *Lm* BCAA-responsive transcriptional regulator capable to directly bind isoleucine, inducing a conformational change that promotes binding to the DNA.^71,72^ This mostly happens under high concentrations of BCAAs, where CodY acts as a repressor of *ilvC* and *purH* genes, involved in BCAAs and purine biosynthesis, respectively.^32,70^ Interestingly, within mammalian cells, where BCAAs are deprived (in particular isoleucine), isoleucine-unbound CodY is capable to activate *prfA* and therefore, PrfA-regulated virulence genes, by directly binding to the first 15 nucleotide of the *prfA*-coding sequence. This suggests an additional functional conformation for CodY in such conditions and thus supports the idea that the gene regulation by CodY depends on its affinity to different sites and the levels of BCAAs available.^70,73^ To this end, *rli60*, a ncRNA encoding a ribosome-mediated transcription attenuator, *cis*-regulates downstream genes involved in BCAA biosynthesis. Under BCAAs limiting conditions, as occurring during infection, the ncRNA *rli60* is transcribed creating a ribosome-mediator attenuator that restricts the expression of the *ivl-leu* operon and consequently the production of BCAA. This BCAA starvation allows *Lm* to control the internal levels of isoleucine thereby promoting virulence gene expression *via* CodY.^32^

## Free fatty acids (FFAs)

After ingestion, *Lm* needs to overcome different metabolomic landscapes throughout the mammalian gastrointestinal tract, with different metabolites produced either by the host itself or by commensal microorganisms or other microbial agents. The ability to sense and respond to these gut metabolites is therefore central for the successful colonization, since it allows bacteria to efficiently regulate gene expression accordingly. Free fatty acids (FFAs) are highly present in the gut metabolome, being important nutrient sources, not only for the resident microbiota but also for bacterial pathogens to signal gene expression by regulating DNA binding activity of virulence-related transcriptional factors.^205^ Furthermore, FFAs act as antimicrobial agents displaying a bacteriostatic or bactericidal activity mainly by targeting the cell membrane.^206^ In *Lm* it was found that the presence of long- or medium- FFAs impairs PrfA-dependent genes activation (Figure 2). FFAs are capable to bind PrfA, inhibiting its DNA binding activity, and preventing the activation of PrfA-regulated virulence genes.^74,75^

## Non-dependent phosphotransferase system host-derived sugars

The host intracellular environment displays different carbon sources that can be used by *Lm*. To import and use these carbon sources, *Lm* has evolved a non-dependent phosphotransferase system (non-dependent PTS) that allow the bacteria to make use of host phosphorylated sugars independently of the PTS system itself, thus offering a more efficient way for sugar utilization.^207,208^ These carbon sources include hexose phosphates, such as glucose-1-phosphate (G1P), glucose-6-phosphate (G6P), fructose-6-phosphate (F6P) and mannose-6-phosphate (M6P), and glycerol, being all signals sensed and rapidly metabolized by *Lm* to improve fitness and growing into the host.^79^

G1P is mainly available intracellularly within the host, as a result of the primary degradation of glycogen.^209^ This sugar can be used by *Lm* intracellularly as a growth substrate, under the positive regulation by PrfA and therefore co-expressed with PrfA-regulated genes in response to diverse host signals.^78^ Beyond G1P, *Lm* is also capable to proficiently import host produced G6P by using Hpt, a non-PTS permease, whose expression is tightly regulated by PrfA (Figure 2). The lack of Htp impairs *Lm* intracytosolic replication and proliferation within mammalian cells, and survival in mice.^79^

Additionally, glycerol can be imported by the *Lm* uptake facilitator GlpF, sustaining its bacterial growth within the host cytosol. In the presence of glycerol, GlpF facilitates its transport across the bacterial cell membrane to be posteriorly metabolized by GlpK, a glycerol kinase that phosphorylates glycerol to produce glycerol-3-phosphate. The resulting product undergoes processing by DhaK kinase, to produce dihydroxyacetone phosphate (DHAP),^210^ which is involved in the glycolysis and gluconeogenesis processes.^211^ Furthermore, the use of glycerol not only provides an advantageous available source of energy, but also controls *Lm* virulence through the upregulation of PrfA-controlled virulence factors such InlC, LLO and ActA, crucial for the bacteria to invade and spread within host cells.^210^

By contrast, other carbon sources such cellobiose and glucose, typically found outside the host, are transported by phosphoenolpyruvate-dependent phosphotransferase systems and may work as signals for *Lm* sensing the surrounding environment, where the PrfA-dependent gene expression is no longer required.^212–214^

## L-glutamine

L-glutamine, an amino acid abundant in mammalian serum and cells, is used by *Lm* as a nitrogen source and was found to be involved in *Lm* virulence modulation (Figure 2).^76,77^ *Lm* can sense the presence of extracellular L-glutamine, using the ABC transporter GlnPQ for its recognition and uptake, GlnP being a substrate binding transmembrane protein, and GLnQ an ATPase.^77^ In the presence of external high concentrations of L-glutamine, GlnPQ senses L-glutamine, inducing the expression of virulence genes (including *hly*, *plcA*, *plcB* and *actA*), and promoting macrophage and mouse infection.^77^ *Lm* has the ability to synthetize L-glutamine itself when it access to both ammonia and glutamate (via glutamate synthase, GnlA).^76^ Yet, the resulting L-glutamine is not enough to induce virulence genes expression. This highlights the ability of *Lm* to distinguish between bacterial or host signals, thus controlling its own localization within the host.^77^

## Reactive oxygen species

Within the human body, bacteria must cope with reactive oxygen species (ROS), such as superoxide (O_2_^−^), hydroxyl radicals (HO) and hydrogen peroxide (H_2_O_2_). Once exposed to ROS, *Lm* can activate specific regulatory pathways that contribute to its survival and virulence within the host. A regulator involved in ROS sensing and gene expression is the transcriptional factor PerR (peroxide-inducible stress response regulator). PerR represses the expression of genes involved in response to peroxide stress and metal homeostasis, including *kat* (hydrogen peroxide decomposition), *fur* (iron homeostasis regulator), *hemA* (heme biosynthesis), *fri* (iron-binding protein) and *fvrA* (iron efflux pump). The absence of *perR* is related with an increased *Lm* sensitivity to H_2_O_2_ *in vitro*, and decreased virulence *in vivo*. This strongly suggests the importance of PerR to both routine peroxide detoxification and response to ROS stress.^87^

ROS generated by activated macrophages, which are fundamental to control *Lm* infection, seem to have also a role in the activation of *Lm* virulence genes *prfA* and *hly*. In activated macrophage-like cells, *hly* expression is enhanced. This increased *hly* expression can be abolished by the addition of superoxide dismutase and catalase, suggesting a role for reactive oxygen intermediates to control *hly* and *prfA* mRNAs levels.^88^ Moreover, mutations leading to the constitutive activation of *prfA* enhance resistance to oxidative stress. Interestingly, the *Lm* resistance to the oxidative stress seems to be dependent on bacterial density.^89^ *Lm* appears thus to be well equipped for both redox sensing and survival during the course of host infection.

## Conclusions and Future Directions

The regulation of virulence factors in the intracellular pathogen *Lm* occurs in a complex and finetuned process, orchestrated by a network of regulatory systems that are able to sense and respond to various signals. These not only include signals secreted by *Lm* itself to promote its fitness and virulence, but also host cues that allow it to recognize the surrounding environment and modulate gene expression accordingly, to support its survival within the host and to facilitate the host colonization. The interplay between *Lm* signaling molecules and the host signals is also necessary to develop a proper response and to adapt to the different host environments.

Importantly, some of these signals that command virulence factors are common to several bacteria, both Gram-positive and Gram-negative. Therefore, deeply knowing and exploring these signals is certainly useful to modulate the expression of bacterial pathogen mechanisms throughout infection. Virulence genes can be essential and thus activated at specific host infection stages to promote virulence, but detrimental for infection at certain point. Furthermore, virulence factors are not expressed homogenously among the entire bacterial population, which per se also constitute a mechanism to optimize infection capacity. Controlling the signals behind these strategies can allow us to modulate pathogenesis, playing with the regulation of virulence genes in time and space, during infection.

Unravelling the bacterial and host signals controlling *Lm* virulence mechanisms facilitates the understanding of its pathogenesis and paves the way for the identification of new potential targets and the design next-generation therapeutic approaches to control *Lm* virulence.
